# Pathohistological Changes in the Gastric Mucosa in Correlation with the Immunohistochemically Detected Spiral and Coccoid Forms of *Helicobacter pylori*

**DOI:** 10.3390/microorganisms12061060

**Published:** 2024-05-24

**Authors:** Nikolina Brkić, Dražen Švagelj, Jelena Omazić

**Affiliations:** 1 Faculty of Medicine, J.J. Strossmayer University of Osijek, 31000 Osijek, Croatia; jelena.omazic@gmail.com; 2 Department of Transfusion Medicine, General County Hospital Vinkovci, 32100 Vinkovci, Croatia; 3 Department of Pathology and Cytology, General County Hospital Vinkovci, 32100 Vinkovci, Croatia; svagelj.pat@gmail.com; 4 Department of Laboratory and Transfusion Medicine, National Memorial Hospital “Dr. Jurjaj Njavro” Vukovar, 32000 Vukovar, Croatia; 5 Department of Medical Chemistry, Biochemistry and Clinical Chemistry, Faculty of Medicine, J.J. Strossmayer University of Osijek, 31000 Osijek, Croatia

**Keywords:** *Helicobacter pylori*, coccoid, chronic gastritis, immunohistochemistry

## Abstract

Background: The coccoid form of *Helicobacter pylori* (*H. pylori)* is resistant to antibiotics. There are only a few studies that have analyzed the frequency of coccoid *H. pylori* in patients with gastritis. The aim of this work was to examine the correlation between the *H. pylori* form and the pathohistological characteristics of the stomach in patients with gastritis. Materials and methods: This research was cross-sectional and focused on the gastric mucosa samples of 397 patients from one general hospital in Croatia. Two independent pathologists analyzed the samples regarding the pathohistological characteristics and the form of *H. pylori*. Results: There was a statistically significant difference in the gender of patients with *H. pylori* gastritis. Only the coccoid form of *H. pylori* was present in 9.6% of patients. There was a statistically significant difference in the frequency of a certain form of the bacterium depending on its localization in the stomach. The intensity of the bacterium was low in the samples where only the coccoid or spiral form was described. In cases of infection in the antrum, premalignant lesions and the coccoid form of *H. pylori* were more often present. Conclusion: In the diagnosis of *H. pylori* infection, the determination of the form of the bacterium via immunohistochemistry should be included to increase the rate of eradication therapy and reduce the incidence of gastric malignancy.

## 1. Introduction

*Helicobacter pylori* (*H. pylori*) was recognized a number of years ago as the cause of chronic inflammation in the human gastric mucosa [1]. Numerous studies have confirmed that neoplasms of the stomach are associated with *H. pylori* infection [2,3,4]. According to the literature, approximately 50.0% of the world’s population is affected by *H. pylori* infection, and its prevalence varies depending on the geographical area and the method used to prove the infection [5]. Most prevalence studies are based on the use of serological methods for *H. pylori* detection and they differ in the populations of developing and developed countries (18.9% to 87.7%) [6]. The current prevalence of active *H. pylori* infection cannot be assessed using serological methods because antibodies to *H. pylori* remain present even after the infection has been cured. In a study conducted on histological preparations of the gastric mucosa where *H. pylori* was detected using an immunohistochemical-specific stain, the frequency of *H. pylori* was 41.0% (in samples from 871 patients in Eastern Croatia) [7].

The previous literature states that, in the stomach, *H. pylori* is found mainly in the spiral form, while, under certain conditions, it can change to a latent (coccoid) form [8,9]. The morphological conversion from the spiral to coccoid form can be induced by any factor that affects the optimal growth conditions, such as reactive oxygen species or the presence of specific pyrimidine nucleotides, as well as the pH, temperature, incubation time, treatment with proton pump inhibitors and treatment with antibiotics. The viability of the coccoid form has been questioned for years. In a mouse model, Cellini et al. showed that the coccoid form of *H. pylori* can cause gastritis [10]. Balakrishna and Filatov described the presence of the coccoid form only in a patient with active gastritis [11]. The coccoid form has a typical bacterial structure—a cell membrane, cytoplasm, and an intact cell wall—thus meeting the criteria for viable forms [12]. Krzyżek and Gościniak hypothesized that the coccoid form of *H. pylori* is responsible for the transmission of the bacterium and the initiation of the infection [13].

In 2017, the World Health Organization included *H. pylori* on the list of antibiotic-resistant pathogens that are a significant threat to human health [14]. Numerous studies show an increase in the number of unsuccessful eradications of *H. pylori*, citing resistance to routinely used antibiotics as the reason [15,16,17,18]. The peptidoglycan layer in the bacterial cell membrane plays an important role in maintaining the shape of the bacterium. Costa et al. assumed that during the transition from the helical to the coccoid form of *H. pylori*, a muropeptide modification occurs in the peptidoglycan layer [19]. According to the research by DeLoney et al., stress signals, including the use of amoxicillin, can stimulate the morphological transition from the spiral form of *H. pylori* to the coccoid form [20]. Drugs and natural compounds that could improve the treatment of *H. pylori* infection are in the research phase [21,22,23,24].

The advantage of the histological method in the diagnosis of gastric mucosa infections is the possibility of grading gastritis according to the Sydney classification—determining the intensity of *H. pylori*, the type and intensity of the inflammation, and premalignant lesions, such as atrophy and metaplasia of the mucosa [25]. The only method for the detection of the form of the bacterium in the gastric mucosa is an *H. pylori*-specific antibody (immunohistochemistry) [26].

The importance of the coccoid form is emphasized in increasing research using machine learning for the detection of both forms [27]. Analyzing the form of bacteria on a histological preparation is extremely tedious and time consuming. Chen et al. presented a machine learning program that can recognize the coccoid and spiral forms of *H. pylori* on a preparation, which will certainly aid pathologists during analysis [27].

In the available literature, there are only a few studies that have analyzed the frequency of the coccoid *H. pylori* form in patients with gastritis [28,29,30,31]. To our knowledge, this is the first work that describes, using the immunohistochemical method, the incidence of the coccoid form in a large number of patients with *H. pylori*-associated gastritis. The aim of this work is to examine the correlation between the coccoid form of *H. pylori* and the histopathological characteristics of the gastric mucosa, thereby aiding in the diagnosis and treatment of *H. pylori* infection. Based on the information about the presence of the coccoid form in the stomach, the clinician might recommend a different approach to treating the infection. Numerous compounds are being investigated that can have an impact on the morphological changes of *H. pylori*. Molecular simulations can be used to analyze the binding of new drugs to the receptors of *H. pylori*, as well as to identify new antigens that could be the target sites for drug binding [23,32].

We believe that the fact that most patients with *H. pylori* gastritis exhibit the coccoid and spiral forms will influence further recommendations for the diagnosis and treatment of the infection.

## 2. Materials and Methods

This research was a cross-sectional study based on archival data from the Department of Pathology and Cytology in one general hospital in Croatia. The materials were tissue samples of the gastric mucosa obtained during the routine gastroscopic examination of 397 patients with dyspeptic symptoms in the period of 1 January 2018 to 31 December 2021. The criteria for the selection of the data and samples were a diagnosis of *H. pylori*-associated gastritis and sufficient gastric mucosal samples (according to the recommendations of the revised Sydney classification, two samples from the antrum, and two samples from the corpus) [25]. Data on age, gender, proton pump inhibitor or H2 receptor antagonist therapy, and previous gastroscopic findings were collected from the medical records. Two independent pathologists analyzed hematoxylin–eosin-stained preparations of the gastric mucosa for the *H. pylori* intensity, the type and intensity of inflammation (granulocyte and mononuclear cells), and the presence and intensity of premalignant lesions (atrophy and metaplasia). The intensity of inflammation and premalignant lesions was described in three degrees: scarce (1), moderate (2), and abundantly present (3). Each gastric mucosa sample was routinely stained using an *H. pylori*-specific immunohistochemical method [33].

Immunohistochemical staining, also used by Tajalli et al. [34], was performed with a concentrated primary antibody, anti-*H. pylori* polyclonal rabbit (DakoCytomation), at a dilution of 1:150 with the Envision Flex antibody diluent (Dako, Glostrup, Denmark), using the Autostainer Link (Dako, Glostrup, Denmark) device for automated immunohistochemical staining. Antigen cells in deparaffinized sections were unmasked by treatment with the PT-Link (109 Dako PT, Glostrup, Denmark) in Target Retrieval Solution, High pH 9. The secondary antibody (streptavidin peroxidase) was used from the set LSAB (Labeled Streptavidin Biotin) + System, HRP (Horseradish Peroxidase) (Dako, Glostrup, Denmark).

On the immunohistochemically stained preparations, the *H. pylori* form was described as spiral only, spiral and coccoid, or coccoid only.

The data were processed in the statistical program Medcalc (MedCalc^®^ Statistical Software version 20.010 (MedCalc Software Ltd., Ostend, Belgium; https://www.medcalc.org; accessed on 1 December 2023)). Nominal variables were compared using Fisher’s exact test. The correlations of the variables were tested with Spearman’s rank correlation coefficient. The level of statistical significance for all comparison tests was *p* < 0.05.

## 3. Results

**Figure 1:**
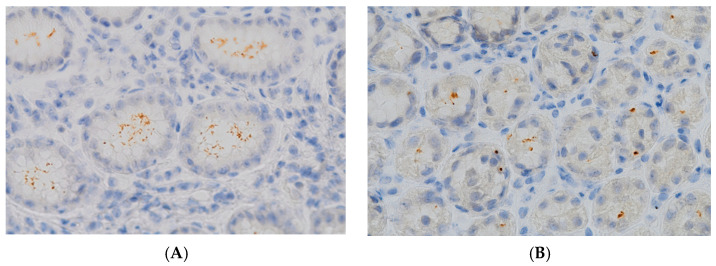
Gastric biopsy specimens showing (**A**) colonization with typical spiral bacilli (IHC, magnification, 600×); (**B**) minimal colonization with atypical coccoid forms (IHC, magnification, 600×).

The spiral and coccoid forms of *H. pylori* detected in the gastric mucosa via the immunohistochemical method are shown in Figure 1.

The median age of the 397 patients was 58 years (range 12–86). There was a statistically significant difference in the gender of patients with *H. pylori* gastritis (Fisher’s exact test, *p* = 0.04). No statistically significant difference was obtained in the frequency of the bacterial forms regarding the gender of patients with *H. pylori* gastritis. Table 1 shows the frequencies of the spiral and coccoid forms of *H. pylori* according to the gender of the patients.

There was no statistically significant difference in the age of patients regarding the form of *H. pylori*. The frequencies of the spiral and coccoid forms by age group are presented in Table 2.

*H. pylori* was present in the antrum samples of 295 patients (74.6%) and in the corpus samples of 387 (97.5%) patients. There was a statistically significant difference in the occurrence of *H. pylori* gastritis depending on its localization in the gastric mucosa (Fisher’s exact test, *p* < 0.001).

Only the coccoid form of *H. pylori* was described in 25 patients (6.3%) in the antrum of the stomach and 13 patients in the body of the stomach (3.3%). There was a statistically significant difference in the frequency of a certain form of the bacterium depending on their localization in the stomach (Fisher’s exact test, *p* = 0.04). The frequencies of the bacterial forms are shown in Table 3.

The intensity of the bacterium was low (1+) in the samples where only the coccoid or spiral form of *H. pylori* was described. The intensity of the bacterium according to its form is presented in Table 4.

The atrophy of the gastric mucosa was present in the antrum in 40 (13.5%) patients and in the corpus in 20 (5.2%) patients. Metaplasia of the gastric mucosa was described in the antrum in 58 (19.6%) patients and the corpus in 27 (7.0%) patients. There was a statistically significant difference in the frequency of premalignant lesions depending on the localization in the stomach (Fisher’s exact test, *p* < 0.001). Table 5 shows the frequencies of the pathohistological characteristics of the gastric mucosa in all patients.

No statistically significant difference was obtained in the pathohistological characteristics of the antrum and corpus of the stomach when only the coccoid or spiral form of the bacterium was present (Table 6 and Table 7).

There was no statistically significant difference in the administration of therapy (proton pump inhibitor or H2 antagonist) before gastroscopy among the groups of patients in whose samples only the spiral or coccoid form was described (Table 8). Most patients from both groups had received their first gastroscopy.

A statistically significant and positive correlation between the intensity of *H. pylori* and the form of the bacterium was obtained in the antrum and corpus samples where both forms were present (Spearman’s correlation coefficient rho 0.78 and 0.4, *p* < 0.001, N = 397). Granulocytic inflammation was positively related to the form and intensity of *H. pylori* in the antrum (Spearman’s correlation coefficient rho 0.35 and 0.44, *p* < 0.001, N = 397).

In cases where only the spiral or coccoid form was present in the antrum, a negative correlation with the intensity of the bacterium was shown (Spearman’s correlation coefficient rho −0.48, *p* = 0.003).

## 4. Discussion

To the best of our knowledge, this research is the first conducted on a large number of human gastric samples of *H. pylori*-associated gastritis, stained with an *H. pylori*-specific immunohistochemical stain. The average age of the 397 patients was 58 years, which is comparable to the studies of patients with gastritis presented by Wen et al. and Nurdin et al. [30,31].

Wen et al. examined the success of *H. pylori* eradication therapy in 433 patients with *H. pylori* chronic active gastritis. After the therapy, 103 patients (23.6%) with a significant response had few bacteria in the coccoid form, with a negative urease breath test. In this group of patients, in the control intervals after 6 months and 1 year, 48.5% and 54.4% were again *H. pylori*-positive [30].

Nurdin et al. immunohistochemically determined only the coccoid form of *H. pylori* in 16 patients (53.3%) with active gastritis [31]. In our study, only the coccoid form was detected in 9.6% of patients; in most patients, both forms of *H. pylori* were described in the gastric mucosa. The intensity of the bacterium was low (1+) in the samples where only the coccoid or spiral form of *H. pylori* was described. Nurdin et al. described the atrophy of the gastric mucosa in 93.3% of cases, which was significantly different from the results of this study. The high frequency of atrophy in the patients could explain the greater proportion of only the coccoid form of the bacterium described by Nurdin et al. [31].

In our study, a negative correlation between the form of *H. pylori* and the intensity of the bacterium was shown (Spearman’s correlation coefficient rho −0.48, *p* = 0.003). There was a statistically significant difference in the presence of atrophy in the corpus depending on the form of *H. pylori* (Fisher’s exact test, *p* < 0.001).

Goldstein et al. found that the incidence of *H. pylori*-associated gastritis was 34.3% (35 out of 109 patients). *H. pylori* is more often present in the antrum of the stomach. The spiral form of *H. pylori* was described only in the gastric mucosa with chronic active inflammation, and the coccoid form was described only in samples with chronic inflammation [28].

Balakrishna et al. presented the case of an 89-year-old patient with *H. pylori*-associated active gastritis and concluded that *H. pylori* causes predominant antral chronic active gastritis and that the coccoid form can cause chronic inflammation of the gastric mucosa [11].

Chan et al. analyzed 111 gastric samples obtained after gastrectomy for adenocarcinoma. The coccoid form was present in 46 (93.9%) of 49 cases of gastric cancer and 7 (50.0%) of 14 cases of peptic ulcers. The coccoid form was present only with the spiral form. Chan et al. concluded that the coccoid form of *H. pylori* colonizes stomachs with adenocarcinoma more frequently than in patients with peptic ulcers [35].

The differences between this research and the literature are that *H. pylori* infection was found to be more common in the corpus (97.5% of corpus samples) and that only samples from patients with *H. pylori* gastritis were analyzed. Both the spiral and coccoid forms were present in most samples.

There was no statistically significant difference in the administration of therapy (proton pump inhibitors or H2 antagonists) before gastroscopy among the groups of patients with only the spiral or coccoid form of *H. pylori*. Considering that the research was cross-sectional and based on historical data, there was a lack of information on the use of antibiotic therapy before gastroscopy. Some of the clinicians provided information about the administration of proton pump inhibitors or H2 antagonists, and these data were analyzed. In cases where it was declared that the patient was on antibiotic therapy, it was not stated which antibiotic was specifically given. We are aware that this is a limitation of our research because it is well described in the literature that certain antibiotics can induce the transition of *H. pylori* into a latent form. In the group of patients with only the spiral or coccoid form, more than 60.0% of the patients were taking proton pump inhibitors, and, for approximately 50.0% of the patients, it was stated that they had received their first gastroscopy. Regarding these patients, we can assume that they did not receive eradication therapy for *H. pylori*, but it cannot be assumed that they did not take antibiotics due to some other inflammatory condition. The impact of antibiotics on the form of *H. pylori* identified in vivo, in the patient’s mucosa, should be investigated in a prospective study.

Gladyshev et al. analyzed the presence of *H. pylori* in patients with gastritis and periodontitis using the immunohistochemical method and detected the bacteria in the oral cavity in both groups. In the gastric mucosa, the spiral form of *H. pylori* dominated, and, in the oral cavity, the coccoid form was more frequent. The authors concluded that the coccoid form of the bacterium should be the target of eradication during the treatment of *H. pylori* infection, which is in line with our conclusions [36].

Using liquid chromatography and mass spectrometry, Loke et al. compared the protein compositions of 3-day-old spiral cells in liquid culture with 3-month-old coccoid cells, from two strains of *H. pylori* (NCTC 11637 and J99), and obtained information indicating that the metabolism of the coccoid form was significantly slowed down in comparison with the spiral cells [37]. Among the proteins that were considerably less present in the coccoid form, thirty-five were enzymes involved in various metabolic pathways, e.g., the metabolism of carbon, amino acids, nucleotides, lipids, vitamins, and cofactors. In contrast, in the coccoid form, the content of 13 metabolic enzymes that ensure long-term survival was increased [37]. The ability to form a biofilm, in which both forms coexist, also indicates the viability of the coccoid form. Moreover, the coccoid form of *H. pylori* has an increased ability to aggregate into monomicrobial bacterial groups surrounded by a thick exopolysaccharide matrix [38].

The treatment of *H. pylori* infection is considered to be the primary method for the prevention of stomach cancer. Amoxicillin (AMX), metronidazole, and proton pump inhibitors are mainly used in the first line of treatment. In a meta-analysis by Savoldi et al. on a large number of clinical isolates, primary resistance to AMX of 10.0% was described [39]. Some studies have shown that antibiotics routinely used for the treatment of *H. pylori* infections, despite being able to induce the transition from the spiral to the coccoid form, do not have a bactericidal effect on the coccoid form [40,41,42]. According to Sarem et al., AMX has the greatest potential to stimulate transformation, while, in vitro, it is most effective against the spiral form of *H. pylori* [40].

Kadkhodaei et al. analyzed the antibiotic resistance of the coccoid and spiral forms of *H. pylori* and found that the coccoid *H. pylori* grew well in successive subcultures in aerobic and microaerobic conditions and produced white spots of mucoid colonies. Both isolates were positive for catalase, oxidase, and urease and contained 16S rDNA. Compared to the spiral *H. pylori*, which was sensitive to almost all antibiotics, the coccoid form was resistant to all antibiotics [43].

It is assumed that the stability of coccoid forms is due to changes in the composition of the cell wall. The importance of the peptidoglycan layer has been demonstrated by the widespread success of antibiotics targeting bacterial cell wall synthesis, such as β-lactams and glycopeptides. From a genome analysis, *H. pylori* appears to have a limited number of enzymes potentially involved in peptidoglycan metabolism in the periplasmic space. There are only three peptidoglycan synthetases, penicillin-binding proteins (PBPs) 1 to 3; two lytic transglycosylases, Slt and MltD; one N-acetylmuramoyl-1-alanine amidase (AmiA); three M23-peptidases, Csd1, Csd2 and Csd3/HdpA; one D,L-endopeptidase, Csd4; and one L,D-endopeptidase, Csd6. *H. pylori* has become a model organism for the study of the selective function of the bacterial cell shape. Chaput et al. concluded that the transition to the coccoid form is regulated by the AmiA protein (N-acetyl-muramoyl-L-alanine-amidase) [44]. A better understanding of the peptidoglycan metabolism in *H. pylori* could lead to new therapeutic strategies [44].

In the last few years, alternative strategies for the treatment of *H. pylori* infection, such as vaccination, nanotechnology, probiotics, and testing of the bacterium’s sensitivity to antibiotics, have been investigated. New diagnostic methods have also been tested, e.g., multiplex PCR, fecal microbiome analysis, and confocal laser endomicroscopy [4].

Several studies have been conducted with natural compounds that have the potential to influence the morphological conversion of *H. pylori*. Bittencourt et al. showed that silibinin affected the morphological conversion of *H. pylori* [45]. Abouwarda et al. compared the binding of fosfomycin and six antibiotics used to treat *H. pylori* infection to the MurA homologous model of *H. pylori* and concluded that fosfomycin had the highest binding affinity for MurA and enhanced the effectiveness of the antibiotics used [46].

Damasceno et al. investigated the antibacterial property of the isocoumarin paepalantine against *H. pylori* and found significant anti-*H. pylori* activity at the minimum inhibitory concentration and the minimum bactericidal concentration [22]. Krzyzek et al. analyzed the influence of myricetin on the morphological transition of *H. pylori* and showed that sub-minimum inhibitory concentrations of myricetin slowed down the process of transition to the coccoid form and the formation of a biofilm. It also enhanced the effects of routinely used antibiotics. They also noted an inhibitory effect of myricetin on the expression of genes involved in the shortening of muropeptide monomers (cds3, csd4, csd6, and amiA) [21].

In other studies, natural compounds that act on the cell wall proteins of some other bacteria have been analyzed, but the same compounds could be applicable to *H. pylori* as well. Mustafa et al. examined the effects of 10 antimicrobial peptides on several multi-resistant bacteria and found that napin strongly binds to penicillin-binding protein 1A of *Acinetobacter baumanii* and snakin-1 interacts with penicillin-binding protein 2 of methicillin-resistant *Staphylococcus aureus* [47]. Rajavel et al. analyzed the effect of the beta-lactam enhancer zidebactam, which inhibits the penicillin-binding proteins of the cell wall of *Pseudomonas aeruginosa* [48].

## 5. Conclusions

In the body of the stomach, 91.4% of patients exhibited both forms (spiral and coccoid) of *H. pylori*. Only the coccoid form of *H. pylori* was present in 9.6% of patients. The infection was more often present in the corpus of the stomach. In cases of infection in the antrum, premalignant lesions of the stomach and the coccoid form of *H. pylori* were more often present. Data on the frequency of infection in different parts of the stomach indicate the importance of taking mucosal samples from several parts of the stomach to reduce the proportion of patients whose results for *H. pylori* infection are falsely negative.

Non-invasive methods and most invasive methods for the detection of *H. pylori* do not provide information on the presence of the coccoid form. The immunohistochemical method specific to *H. pylori* detects both the spiral and coccoid forms. Given that the coccoid form of the bacterium can only be distinguished from inflammatory cells and some other bacteria using the immunohistochemical method, we emphasize the importance of choosing an immunohistochemical method for the detection of *H. pylori* infection.

We hypothesize that the coccoid form is the reason for the failure of eradication therapy, and we propose that more attention be paid to the methods of eradicating the coccoid form. We believe that it is important to research drugs and new compounds that affect the proteins of the cell wall of *H. pylori* in order to prevent the morphological transition to the latent coccoid form, which is resistant to most known antibiotics.

Based on the above conclusions, we suggest that, in the diagnosis of *H. pylori* infection, the determination of the form of the bacterium using the immunohistochemical method should be included to increase the success of eradication therapy and thereby reduce the risk of premalignant and malignant lesions forming in the gastric mucosa.

## Figures and Tables

**Table 1:** Frequencies of spiral and coccoid *H. pylori* according to gender of patients.

Patients	Male N (%)	Female N (%)	*p* *
All	185 (100.0)	215 (100.0)	0.04
With Coccoid Only	15 (8.1)	23 (10.7)	0.1
With Spiral Only	8 (4.3)	11 (5.1)	0.5
Spiral + Coccoid	162 (87.6)	181 (84.2)	0.2

* Fisher’s exact test.

**Table 2:** Frequencies of spiral and coccoid forms by age group.

Age Group (Years)	Coccoid Only N (%)	Spiral Only N (%)	*p* *
<20	1 (2.6)	0 (0.0)	1.0
21–40	4 (10.5)	3 (15.8)	0.7
41–60	16 (42.1)	9 (47.4)	0.8
>60	17 (44.7)	7 (36.8)	0.8
Total (N/%)	38 (100.0)	19 (100.0)	N/A

* Fisher’s exact test; N/A, not applicable.

**Table 3:** Frequencies of *H. pylori* form in antrum and corpus in all patients.

*H. pylori* Form	Antrum N (%)	Corpus N (%)	*p* *
Spiral	10 (2.5)	11 (2.8)	0.8
Spiral + Coccoid	261 (65.7)	363 (91.4)	<0.001
Coccoid	25 (6.3)	13 (3.3)	0.04
No *H. pylori*	101 (25.4)	10 (2.5)	<0.001
Total	397 (100.0)	397 (100.0)	N/A

* Fisher’s exact test; N/A, not applicable.

**Table 4:** Intensity of *H. pylori* in the antrum and corpus in all patients.

Intensity	Antrum N (%)			Corpus N (%)		
	Spiral	Spiral + Coccoid	Coccoid	Spiral	Spiral + Coccoid	Coccoid
1	7 (70.0)	64 (24.6)	25 (100.0)	8(72.7)	76 (20.9)	13 (100.0)
2	2 (20.0)	107 (41.2)	0 (0.0)	2 (18.2)	160 (44.1)	0 (0.0)
3	1 (10.0)	90 (34.6)	0 (0.0)	1 (9.0)	127 (35.0)	0 (0.0)
total	10 (100.0)	261 (100.0)	25 (100.0)	11 (100.0)	363 (100.0)	13 (100.0)

**Table 5:** Pathohistological characteristics of gastric mucosa in all *H. pylori*-positive patients.

Variable	Antrum N (%)		Corpus N (%)		*p* *
	Yes	No	Yes	No	
Granulocyte	238 (80.4)	58 (19.6)	325 (84.0)	62 (16.0)	0.22
Mononuclear cells	296 (100.0)	0 (0.0)	386 (99.7)	1 (0.3)	1.0
Atrophy	40 (13.5)	256 (86.5)	20 (5.2)	367 (94.8)	<0.001
Metaplasia	58 (19.6)	238 (80.4)	27 (7.0)	360 (93.9)	<0.001
Total	296 (100.0)		387 (100.0)		

* Fisher’s exact test.

**Table 6:** Pathohistological characteristics of antrum depending on *H. pylori* form.

Variable		Spiral + Coccoid N (%)	Coccoid Only N (%)	Spiral Only N (%)	*p* *
Granulocyte	No	49 (18.8)	7 (28.0)	2 (20.0)	1.0
	Yes	212 (81.2)	18 (72.0)	8 (80.0)	1.0
Mononuclear cells	No	0 (0.0)	0 (0.0)	0 (0.0)	N/A
	Yes	261 (100.0)	25 (100.0)	10 (100.0)	N/A
Atrophy	No	226 (86.6)	22 (88.0)	8 (80.0)	0.6
	Yes	35 (13.4)	3 (12.0)	2 (20.0)	0.6
Metaplasia	No	211 (80.8)	19 (78.0)	8 (80.0)	1.0
	Yes	50 (19.2)	6 (24.0)	2 (20.0)	1.0
Total		261 (100.0)	25 (100.0)	10 (100.0)	N/A

* Fisher’s exact test between coccoid and spiral only; N/A, not applicable.

**Table 7:** Pathohistological characteristics of corpus depending on *H. pylori* form.

Variable		Spiral + Coccoid N (%)	Coccoid Only N (%)	Spiral Only N (%)	*p* *
Granulocyte	No	53 (14.6)	7 (53.8)	2 (18.2)	0.1
	Yes	310 (85.4)	6 (46.2)	9 (81.7)	0.1
Mononuclear cells	No	1 (0.3)	0 (0.0)	0 (0.0)	N/A
	Yes	362 (97.7)	13 (100.0)	10 (100.0)	N/A
Atrophy	No	345 (95.0)	11 (84.6)	11 (100.0)	0.5
	Yes	18 (5.0)	2 (15.5)	0 (0.0)	0.5
Metaplasia	No	342 (93.9)	9 (69.2)	10 (90.9)	0.3
	Yes	22 (6.1)	4 (30.8)	1 (9.1)	0.6
Total		363 (100.0)	13 (100.0)	11 (100.0)	N/A

* Fisher’s exact test between coccoid and spiral only; N/A, not applicable.

**Table 8:** Therapy in patients with spiral and coccoid-only forms of *H. pylori*.

Variable	Therapy before Gastroscopy N (%)		Gastroscopy N (%)		
	Yes	No	First	Control	No Data
Coccoid only N = 38	25 (65.8)	13 (34.2)	20 (52.6)	5 (13.2)	13 (34.2)
Spiral only N = 19	13 (68.4)	6 (31.6)	12 (63.2)	2 (10.5)	5 (26.3)
*p* *	1.0	1.0	0.6	1.0	0.8

* Fisher’s exact test.

## Data Availability

The raw data supporting the conclusions of this article will be made available by the authors upon request.

## References

[1] Marshall B.J., Warren J.R. (1984). Unidentified Curved Bacilli in the Stomach of Patients with Gastritis and Peptic Ulceration. Lancet.

[2] Altayar O., Davitkov P., Shah S.C., Gawron A.J., Morgan D.R., Turner K., Mustafa R.A. (2020). AGA Technical Review on Gastric Intestinal Metaplasia—Epidemiology and Risk Factors. Gastroenterology.

[3] Sotelo O.C., Rojas M.P., Rodríguez A.R., Figueroa V.S., Jofré R.A., Bufadel Godoy M.E., González P.C., Donoso R.G., López E.F., Selvat G.L. Estrategias Para La Prevención Primaria y Secundaria Del Cáncer Gástrico: Consenso Chileno de Panel de Expertos Con Técnica Delfi. Gastroenterol. Hepatol..

[4] Ali A., AlHussaini K.I. (2024). Helicobacter Pylori: A Contemporary Perspective on Pathogenesis, Diagnosis and Treatment Strategies. Microorganisms.

[5] Malfertheiner P., Megraud F., Rokkas T., Gisbert J.P., Liou J.M., Schulz C., Gasbarrini A., Hunt R.H., Leja M., O’Morain C. (2022). Management of Helicobacter Pylori Infection: The Maastricht VI/Florence Consensus Report. Gut.

[6] Hooi J., Lai W., Ng W.K., Suen M.M.Y., Underwood F.E., Tanyingoh D., Malfertheiner P., Graham D.Y., Wong V.W.S., Wu J.C.Y. (2017). Global Prevalence of Helicobacter Pylori Infection: Systematic Review and Meta-Analysis. Gastroenterology.

[7] Brkic N., Terzic V., Švagelj M., Cvrkovic M., Brkic H., Švagelj D. (2017). The Prevalence and Characteristics of Helicobacter Pylori-Associated Gastritis in Dyspeptic Patients in Eastern Croatia, Determined by Immunohistochemistry. Period. Biol..

[8] Gladyshev N., Taame M., Kravtsov V. (2022). Helicobacter Pylori Coccoid Forms as a Possible Target of Eradication Therapy. Infect. Disord. Drug Targets.

[9] Ierardi E., Losurdo G., Mileti A., Paolillo R., Giorgio F., Principi M., Di Leo A. (2020). The Puzzle of Coccoid Forms of Helicobacter Pylori: Beyond Basic Science. Antibiotics.

[10] Cellini L., Allocati N., Angelucci D., Iezzi T., Di Campli E., Marzio L., Dainelli B. (1994). Coccoid Helicobacter Pylori Not Culturable in Vitro Reverts in Mice. Microbiol. Immunol..

[11] Balakrishna J.P., Filatov A. (2013). Coccoid Forms of Helicobacter Pylori Causing Active Gastritis. Am. J. Clin. Pathol..

[12] Reshetnyak V.I., Reshetnyak T.M. (2017). Significance of Dormant Forms of Helicobacter Pylori in Ulcerogenesis. World J. Gastroenterol..

[13] Krzyzek P., Gosciniak G. (2018). A Proposed Role for Diffusible Signal Factors in the Biofilm Formation and Morphological Transformation of Helicobacter Pylori. Turk. J. Gastroenterol..

[14] Tacconelli E., Carrara E., Savoldi A., Harbarth S., Mendelson M., Monnet D.L., Pulcini C., Kahlmeter G., Kluytmans J., Carmeli Y. (2018). Discovery, Research, and Development of New Antibiotics: The WHO Priority List of Antibiotic-Resistant Bacteria and Tuberculosis. Lancet Infect. Dis..

[15] Al-Fakhrany O.M., Elekhnawy E. (2023). Helicobacter Pylori in the Post-Antibiotics Era: From Virulence Factors to New Drug Targets and Therapeutic Agents. Arch. Microbiol..

[16] Lin Y., Shao Y., Yan J., Ye G. (2023). Antibiotic Resistance in Helicobacter Pylori: From Potential Biomolecular Mechanisms to Clinical Practice. J. Clin. Lab. Anal..

[17] Li M., Ma X., Xu H., Han M., Gou L., Du H., Wei L., Zhang D. (2024). Assessment of the Quality, Diagnosis, and Therapeutic Recommendations of Clinical Practice Guidelines on Patients with Helicobacter Pylori Infection: A Systematic Review. Gastroenterol. Hepatol..

[18] Chen C.L., Wu I.T., Wu D.C., Lei W.Y., Tsay F.W., Chuah S.K., Chen K.Y., Yang J.C., Liu Y.H., Kuo C.H. (2023). Independent Risk Factors Predicting Eradication Failure of Hybrid Therapy for the First-Line Treatment of Helicobacter Pylori Infection. Microorganisms.

[19] Costa K., Bacher G., Allmaier G., Dominguez-Bello M.G., Engstrand L., Falk P., de Pedro M.A., Portillo F.G.-D. (1999). The Morphological Transition of Helicobacter Pylori Cells from Spiral to Coccoid Is Preceded by a Substantial Modification of the Cell Wall. J. Bacteriol..

[20] DeLoney C.R., Schiller N.L. (1999). Competition of Various β-Lactam Antibiotics for the Major Penicillin-Binding Proteins of Helicobacter Pylori: Antibacterial Activity and Effects on Bacterial Morphology. Antimicrob. Agents Chemother..

[21] Krzyżek P., Migdał P., Paluch E., Karwańska M., Wieliczko A., Gościniak G. (2021). Myricetin as an Antivirulence Compound Interfering with a Morphological Transformation into Coccoid Forms and Potentiating Activity of Antibiotics against Helicobacter Pylori. Int. J. Mol. Sci..

[22] Damasceno J.P.L., Rodrigues R.P., Gonçalves R.D.C.R., Kitagawa R.R. (2017). Anti-Helicobacter Pylori Activity of Isocoumarin Paepalantine: Morphological and Molecular Docking Analysis. Molecules.

[23] González A., Salillas S., Velázquez-Campoy A., Espinosa Angarica V., Fillat M.F., Sancho J., Lanas Á. (2019). Identifying Potential Novel Drugs against Helicobacter Pylori by Targeting the Essential Response Regulator HsrA. Sci. Rep..

[24] Silvan P., Moreno J.M., Carvajal D.A., Martinez-Rodriguez M., Garcia-Ibañez P., Silvan J.M., Moreno D.A., Carvajal M., Martinez-Rodriguez A.J. (2023). Influence of Source Materials, Concentration, Gastric Digestion, and Encapsulation on the Bioactive Response of Brassicaceae-Derived Samples against Helicobacter Pylori. Microorganisms.

[25] Dixon M.F., Genta R.M., Yardley J.H., Correa P. (1996). Classification and Grading of Gastritis: The Updated Sydney System. Am. J. Surg. Pathol..

[26] Akeel M., Elhafey A., Shehata A., Elmakki E., Aboshouk T., Ageely H., Mahfouz M.S. (2021). Efficacy of Immunohistochemical Staining in Detecting Helicobacter Pylori in Saudi Patients with Minimal and Atypical Infection. Eur. J. Histochem..

[27] Zhong Z., Wang X., Li J., Zhang B., Yan L., Xu S., Chen G., Gao H. (2022). A Study on the Diagnosis of the Helicobacter Pylori Coccoid Form with Artificial Intelligence Technology. Front. Microbiol..

[28] Goldstein N.S. (2002). Chronic Inactive Gastritis and Coccoid Helicobacter Pylori in Patients Treated for Gastroesophageal Reflux Disease or with H Pylori Eradication Therapy. Am. J. Clin. Pathol..

[29] Aggarwal N., Snyder P., Owens S.R. (2011). Unusual Helicobacter Pylori in Gastric Resection Specimens: An Old Friend with a New Look. Int. J. Surg. Pathol..

[30] Wen M., Zhang Y., Yamada N., Matsuhisa T., Matsukura N., Sugisaki Y. (1999). An Evaluative System for the Response of Antibacterial Therapy: Based on the Morphological Change of Helicobacter Pylori and Mucosal Inflammation. Pathol. Int..

[31] Nurdin W., Krisnuhoni E., Kusmardi K. (2016). Comparison of Helicobacter Pylori Detection Using Immunohistochemistry and Giemsa and Its Association with Morphological Changes in Active Chronic Gastritis. Indones. J. Gastroenterol. Hepatol. Dig. Endosc..

[32] Attaran B., Salehi N., Ghadiri B., Esmaeili M., Kalateh S., Tashakoripour M., Eshagh Hosseini M., Mohammadi M. (2021). The Penicillin Binding Protein 1A of Helicobacter Pylori, Its Amoxicillin Binding Site and Access Routes. Gut Pathog..

[33] Lash R.H., Genta R.M. (2016). Routine Anti-Helicobacter Immunohistochemical Staining Is Significantly Superior to Reflex Staining Protocols for the Detection of Helicobacter in Gastric Biopsy Specimens. Helicobacter.

[34] Tajalli R., Nobakht M., Mohammadi-Barzelighi H., Agah S., Rastegar-Lari A., Sadeghipour A. (2013). The Immunohistochemistry and Toluidine Blue Roles for Helicobacter Pylori Detection in Patients with Gastritis. Iran. Biomed. J..

[35] Chan W.-Y., Hui P.-K., Leung K.-M., Chow J., Kwok F., Ng C.-S. (1994). Coccoid Forms of Helicobacter Pylori in the Human Stomach. Am. J. Clin. Pathol..

[36] Gladyshev N., Taame M., Ibiliev A., Grukhin Y., Kravtsov V. (2022). Colonization by Various Morphological Forms of Helicobacter Pylori in the Gingival Sulcus and Antrum of the Stomach. Recent Adv. Anti-Infect. Drug Discov..

[37] Loke M.F., Ng C.G., Vilashni Y., Lim J., Ho B. (2016). Understanding the Dimorphic Lifestyles of Human Gastric Pathogen Helicobacter Pylori Using the SWATH-Based Proteomics Approach. Sci. Rep..

[38] Cellini L., Grande R., Di Campli E., Traini T., Di Giulio M., Nicola Lannutti S., Lattanzio R. (2008). Dynamic Colonization of Helicobacter Pylori in Human Gastric Mucosa. Scand. J. Gastroenterol..

[39] Savoldi A., Carrara E., Graham D.Y., Conti M., Tacconelli E. (2018). Prevalence of Antibiotic Resistance in Helicobacter Pylori: A Systematic Review and Meta-Analysis in World Health Organization Regions. Gastroenterology.

[40] Sarem M., Corti R. (2016). Role of Helicobacter Pylori Coccoid Forms in Infection and Recrudescence. Gastroenterol. Y Hepatol. (Engl. Ed.).

[41] Faghri J., Poursina F., Moghim S., Esfahani H.Z., Esfahani B.N., Fazeli H., Mirzaei N., Jamshidian A., Safaei H.G. (2014). Morphological and Bactericidal Effects of Different Antibiotics on Helicobacter Pylori. Jundishapur J. Microbiol..

[42] Gladyshev N., Taame M., Kravtsov V. (2020). Clinical and Laboratory Importance of Detecting Helicobacter Pylori Coccoid Forms for the Selection of Treatment. Gastroenterol. Rev. Przegląd Gastroenterol..

[43] Kadkhodaei S., Siavoshi F., Noghabi K.A. (2020). Mucoid and Coccoid Helicobacter Pylori with Fast Growth and Antibiotic Resistance. Helicobacter.

[44] Chaput C., Ecobichon C., Pouradier N., Rousselle J.-C., Namane A., Boneca I.G. (2016). Role of the N-Acetylmuramoyl-l-Alanyl Amidase, AmiA, of Helicobacter Pylori in Peptidoglycan Metabolism, Daughter Cell Separation, and Virulence. Microb. Drug Resist..

[45] Bittencourt M.L.F., Rodrigues R.P., Kitagawa R.R., Gonçalves R. (2020). de C.R. The Gastroprotective Potential of Silibinin against Helicobacter Pylori Infection and Gastric Tumor Cells. Life Sci..

[46] Abouwarda A.M., Ismail T.A., Abu El-Wafa W.M., Faraag A.H.I. (2022). Synergistic Activity and Molecular Modelling of Fosfomycin Combinations with Some Antibiotics against Multidrug Resistant Helicobacter Pylori. World J. Microbiol. Biotechnol..

[47] Mustafa G., Mehmood R., Mahrosh H.S., Mehmood K., Ahmed S. (2022). Investigation of Plant Antimicrobial Peptides against Selected Pathogenic Bacterial Species Using a Peptide-Protein Docking Approach. Biomed. Res. Int..

[48] Rajavel M., Kumar V., Nguyen H., Wyatt J., Marshall S.H., Papp-Wallace K.M., Deshpande P., Bhavsar S., Yeole R., Bhagwat S. (2021). Structural Characterization of Diazabicyclooctane β-Lactam “Enhancers” in Complex with Penicillin-Binding Proteins PBP2 and PBP3 of Pseudomonas Aeruginosa. mBio.

